# The utility of adaptive eLearning data in predicting dental students’ learning performance in a blended learning course

**DOI:** 10.5116/ijme.64f6.e3db

**Published:** 2023-10-06

**Authors:** Farhan H. Alwadei, Blasé P. Brown, Saleh H. Alwadei, Ilene B. Harris, Abdurahman H. Alwadei

**Affiliations:** 1 Department of Preventive Dental Sciences, College of Dentistry, Prince Sattam Bin Abdulaziz University, Al Kharj, Saudi Arabia; 2 Department of Oral Medicine and Diagnostic Sciences, College of Dentistry, University of Illinois at Chicago, Chicago, Illinois, USA; 3 Department of Medical Education, Department of Pathology, Department of Curriculum and Instruction, Curriculum Studies with Emphasis on Health Professions Education, College of Medicine, College of Education, University of Illinois at Chicago, Chicago, Illinois, USA; 4 Department of Pediatric Dentistry and Orthodontics, College of Dentistry, King Saud University, Riyadh, Saudi Arabia

**Keywords:** Adaptive learning, learning analytics, adaptive learning analytics, self-regulated learning, educational technology, computer-assisted instruction

## Abstract

**Objectives:**

To examine the impact of dental students’ usage patterns within an adaptive learning platform (ALP), using ALP-related indicators, on their final exam performance.

**Methods:**

Track usage data from the ALP, combined with demographic and academic data including age, gender, pre- and post-test scores, and cumulative grade point average (GPA) were retrospectively collected from 115 second-year dental students enrolled in a blended learning review course. Learning performance was measured by post-test scores. Data were analyzed using correlation coefficients and linear regression tests.

**Results:**

The ALP-related variables (without controlling for background demographics and academic data) accounted for 29.6% of student final exam performance (R^2^=0.296, F_(10,104)_=4.37, p=0.000). Positive significant ALP-related predictors of post-test scores were improvement after activities (β=0.507, t_(104)_=2.101, p=0.038), timely completed objectives (β=0.391, t_(104)_=2.418, p=0.017), and number of revisions (β=0.127, t_(104)_=3.240, p=0.002). Number of total activities, regardless of learning improvement, negatively predicted post-test scores (β= -0.088, t_(104)_=-4.447, p=0.000). The significant R^2^ change following the addition of gender, GPA, and pre-test score (R^2^=0.689, F_(13, 101)_=17.24, p=0.000), indicated that these predictors explained an additional 39% of the variance in student performance beyond that explained by ALP-related variables, which were no longer significant. Inclusion of cumulative GPA and pre-test scores showed to be the strongest and only predictors of post-test scores (β=18.708, t_(101)_=4.815, p=0.038) and (β=0.449, t_(101)_=6.513, p=0.038), respectively.

**Conclusions:**

Track ALP-related data can be valuable indicators of learning behavior. Careful and contextual analysis of ALP data can guide future studies to examine practical and scalable interventions.

## Introduction

Recently, there has been a rapid growth of adaptive learning platforms (ALP) in the education marketplace due to technological advances.^1^ These platforms react and “adapt” to individual student input. To understand how an ALP can be designed, implemented, and evaluated effectively, it is necessary to understand the behavior patterns students with different attributes exhibit when interacting within the ALP and how these behavior patterns might shed light on learning outcomes.^2^^, ^^3^ As students interact with an ALP, they leave digital traces of their work, such as logging in and out, materials viewed or downloaded, time spent, number of attempts on assignments/tests, and interactions with peers/instructors. Traces of students’ efforts collected across the timeline of a course can explain valuable learning behaviors.^4^

According to the Society for Learning Analytics Research, learning analytics (LA) is “the measurement, collection, analysis and reporting of data about learners and their contexts for purposes of understanding and optimizing learning and the environment in which it occurs”.^5^ To this end, LA is used to provide informative feedback to students and instructors and to develop predictive models for anticipating academic success, identifying at-risk students, and providing actionable recommendations for targeted interventions.^5^ In the emerging field of LA, track data from non-adaptive learning management systems (LMS) constitute the main data source.^4^

Valuable insights can be obtained about students’ learning processes as they interact with adaptive platforms, through the measurement, collection, analysis, and reporting of multi-modal data, which is referred to as adaptive learning analytics.^6^ In their systematic review, Mavroudi and colleagues (2018), focused on the synergic relationship between adaptive learning and LA and demonstrated how combining both domains not only address learners’ variability and diversity, but also serves as a promising research approach, using analytics to investigate data sources from adaptive systems.^6^ Others have highlighted the important contribution of LA to the development of ALPs.^7^^-^^9^

The foundational framework guiding this research is a synthesis of theoretical and practical LA models, adaptive learning, self-regulated learning, and digital learning in an adaptive environment. A common key feature across theoretical LA research is the idea that self-regulated learners actively participate in their own learning processes, using metacognitive, behavioral, and motivational self-management strategies to achieve their goals.^10^ The well-understood impact of self-regulation in learning on student behavior and outcomes is observed within an adaptive digital environment, where adaptive feedback and student self-regulation work synergistically.^6^^, ^^11^

To the best of the authors’ knowledge, the interplay between LA and learning in an adaptive digital environment has not been explored on a large scale in the context of health professions education. The overall purpose of this study is to determine whether automatically generated student activity and outcome data in a specific ALP are associated with, and predictive of, dental students' academic achievement in a single 4-week preparatory review course for the National Board Dental Examination (NBDE), controlling for demographic and prior academic-related variables. Specifically, we aim to explore the relationship between student usage patterns within the ALP and their learning performance on the final exam; and to assess the impact of ALP-related variables on the likelihood of success on the final exam, controlling for gender and prior academic performance (i.e., cumulative GPA and pre-test scores).

## Methods

The University of Illinois at Chicago institutional review board reviewed and approved the study. The data were retrospectively obtained from a convenience sample of second-year dental students who were enrolled in a single course offered in a blended learning format during the academic years 2016-2018, where an ALP was used, and its online homework grades constituted 30–35% of the cumulative grade in the course. A total of 115 students represents the entire study population. Detailed tracking logs from the ALP present the study's largest and most novel data source (Table 1). We collected 15 tracking indicators from the ALP as independent variables (Table 1). Additional demographics and background academic information were collected including average age, gender, pre-test scores, cumulative GPA prior to course enrollment, and course grade after course completion (Table 2).

Students’ knowledge of the course content was measured by electronically administered written pre-/posttests, which comprised different formats including multiple choice, true/false, and matching questions. Specificity and reliability of these tests were addressed via careful test construction by an experienced course instructor with input from other content experts from different subjects, and by aligning the test items with development guidelines provided by the Joint Commission on National Dental Examinations. The pre-tests were conducted formatively one week prior to course enrollment. Final exam raw scores were used as a measure of students' academic success (i.e., dependent variable). In this study, the terms “final exam” and “post-test” are used interchangeably.

The ALP (i.e., education intervention) was incorporated into a review preparatory course for the national board dental examination to provide students with targeted and efficient review process and improve their learning outcomes. Thus, the course was redesigned from a face-to-face format to a blended learning format. During the academic years 2016-2018, the ALP was implemented summatively and included 35 learning objectives under 4 overarching learning modules including: (1) Anatomic Sciences; (2) Biochemistry, Dental Anatomy, and Oral Pathology; (3) Microbiology-Immunology; and (4) General Pathology. In addition to text-based content, the ALP included different learning resources such objective test questions, exercises, case studies, videos, and audios. The course instructor formulated the learning objectives with matching content and practice/test questions within each learning module. Guided by specific learning objectives, the learning module contains various number of learning activities and assessment questions. As students interact with the ALP, to demonstrate mastery of learning objectives within a specific-time period (i.e., due dates), the ALP selects the most appropriate pedagogical content and assessment (i.e., learning experience) based on real-time analysis of different metrics displayed by individual students.^12^

**Table 1:** The adaptive learning platform (ALP) indicators (independent variables)

ALP measures	Operational definition and contextual relationship to self-regulated learning
Diagnostic test performed	- The percentage of diagnostic tests completed by the student before learning modules. In the ALP, diagnostic tests are referred to as Determine Knowledge operation. - A proxy for Self-awareness (conscientiousness) and effective learning strategy (i.e., using the Determine Knowledge operation to guide the learning process).
Knowledge state	- The average achievement that students accomplish in a module via assessment exercises. - An outcome measure that will indirectly impact other variables in relation to SRL contextualization (e.g., self-awareness, engagement, and effort/motivation).
Knowledge covered	- The percentage of topics (topics’ entire content) completed for the course modules. Content within a topic could include a variety of instructional resources, such as text, animations, practice tests, case studies, assignments, postings, and discussions. If students cover all topic activities related to a module within the ALP, the system documents 100% for percentage of topics completed. - A proxy for students’ engagement with the ALP course content.
Completed objectives	- Percentage of learning objectives completed, regardless of the due date. - A proxy for self-monitoring (conscientiousness) and organization (time management skills).
Timely completed objectives	Percentage of learning objectives completed before the due date. - A proxy for self-monitoring and organization (more mature time organization strategies).
Number of practice operations	Total number of assessment questions at the objective level with a recorded value, independent of those where students practiced initially during their first exposure to the learning modules. - A proxy for effort (persistent behavior) or effective learning strategy (following the ALP algorithmic suggestions to practice more).
Number of revisions	Total of the revised operations with a recorded value for which student’s returned to previously completed lessons and assessments questions. - A proxy for effort/motivation (persistent behavior) or effective learning strategy (following the ALP algorithmic suggestions to perform more revision).
Number of lessons	- Number of lessons on new learning objectives that were started by the student. - A proxy for engagement or effective learning strategy (i.e., using the Determine Knowledge operation to provide targeted content).
Total activities	- Total number of lessons that were started, along with practice and determine knowledge operations. - An encompassing proxy for engagement with the ALP course content.
Average score	- Average score for the contributing activities, from lessons or assessment operations. - An outcome measure that will indirectly impact other variables in relation to SRL contextualization (e.g., self-awareness, engagement, and effort/motivation)
Improvement after activities	- Percentage of recorded activities that led to an improvement (i.e., lessons that demonstrated growth/increase in student’s knowledge state level. - A proxy for effective learning strategy or productive struggle.
Number of assessments	- Number of questions that were accessed, attempted and/or completed by the student. - A proxy for engagement and effort.
Correctly answered assessments	Percentage of questions that were marked correct. - A proxy for engagement.
Incorrectly answered assessments	- Percentage of questions where the provided answers were wrong, incomplete, or for which no answer was supplied. - A proxy for effort (persistent behavior).
Total time	- Total number of minutes that were spent in lessons or practice/determined knowledge operations. - A proxy for engagement with the ALP course content.

This study utilizes the application of data analytics methodologies and strategies (i.e., collection of vast amounts of data for retrospective analysis and reports). In addition to real-time data integration and application to support individualized learning as described above, the ALP has been built to collect deep granular learner data that are available for any retrospective analysis, referred to as Analytical Reviews.^13^

Through various tools, the ALP can provide manual and automated generation of reports and analytics. At the request of stakeholders (i.e., course instructors, researchers, or institution leaders) the ALP engineers can automatically provide all raw data, especially those of interest to stakeholders, which impact the learning process including curriculum, content, learners, and instructors. The data collected for this study was informed by the theory of self-regulation of learning, practical recommendations described in previous studies,^11^^,^^14^ and consensus among the authors after discussions with the course director and principal researcher for the ALP. As clarified in Table 1, these data represent a family of behaviors that reflect subsets of self-regulated learning, such as effective and efficient learning strategies, self-awareness, self-monitoring, effort (persistent behavior), motivation, engagement, and time-management skills.

**Table 2:** Demographic and academic information as well as learning activity and outcome within the adaptive platform

Variable	Mean (%) ± SD
Average age	24
Gender (female)	63 (64.2%)
Grades earned	
Excellent (A)	50 (42.7%)
Good (B)	62 (53.0%)
Passing (C)	5 (4.3%)
Cumulative GPA	3.49 ± .37
Tests’ scores	
Pre-test	122.9 ± 20.6
Post-test (final exam)	148.4 ± 17.7
Adaptive Learning Measures	
Knowledge state	91.82 ± 4.28
Knowledge covered	99.03 ± 2.90
Average score	87.07 ± 5.48
Completed objectives	96.92 ± 5.19
Timely completed objectives	85.76 ± 13.47
Determine knowledge performed	71.77 ± 27.09
Total number of questions	1445 ± 384.34
Incorrectly answered question	20.21 ± 4.71
Correctly answered question	79.79 ± 4.71
Number of lessons	198.24 ± 60.37
Total activities	540.94 ± 189.32
Improvement after activities	78.22 ± 8.06
Number of practice operations	46.97 ± 61.16
Number of revisions	108.09 ± 77.59
Total time (minutes)	2530.69 ± 1782.29

Data was managed using Microsoft Excel and imported into SPSS statistical software to conduct data analyses. Pearson correlation coefficients were conducted to analyze the relationship between final exam scores and other variables (demographic, academic, and ALP-related). When evaluating the strength of correlation, the following classification was used: strong if ρ is > 0.7 and ≤ 1.0, moderate if ρ is ≥ 0.4 and ≤ 0.7, and weak if ρ is > 0.2 and < 0.4. Linear regression tests were used to assess the impact of identified ALP-related variables on the likelihood of success on the final exam, controlling for gender and prior academic performance (i.e., cumulative GPA and pre-test scores). Several ALP-related measures were omitted from linear regression models because of their collinearity with other target variables.

**Table 3:** Significant bivariate correlation of final exam scores with the adaptive platform activity and outcome measures (n = 115)

Bivariate correlations with final exam scores	Coefficients of correlation	p value
Knowledge state	r = .32	0.000
Knowledge covered	r = .20	0.016
Timely completed objectives	r = .17	0.035
Total activities	r = - .21	0.012
Improvement after activities	r = .27	0.002
Incorrect assessment	r = - 36	0.000
Correct assessment	r = 36	0.000
Number of lessons	r = - 19	0.017
Cumulative GPA	r = .71	0.000
Pre-test scores	r = .76	0.000

The fully fitted model allowed for the selection of variables with the “best” subset of predictors of students’ academic performance on the final exam. Statistical significance was noted at p value < 0.05.

## Results

Table 2 displays descriptive statistics for students’ demographic and academic information as well as students’ learning activity and outcome data demonstrated by various ALP measures. Pearson correlation coefficients showed a range of significant (small positive) correlations between final exam scores and several of the ALP activity and outcome measures (r_(__113)_ = 0.17 to 0.358, p= 0.000 to 0.035) (Table 3). Contrary to expectations, negative and statistically significant correlations between final exam scores and total number of activities and number of lessons (r_(__113)_= -0.21, p= 0.012; and r_(113)_ = -0.192, p= 0.017, respectively). Background academic data, reflected by cumulative GPA and pre-test scores, had strong positive and statistically significant correlations with final exam scores (r_(__113)_ = 0.71 and 0.76 respectively, p= 0.000).

Multiple linear regression analyses were used to predict student performance on the final exam based on ALP-related indicators and background demographics and academic data. As displayed in Table 4, the ALP-related variables (without controlling for background demographics and academic data) accounted for 29.6% of final exam performance (R^2^= 0.296, F_(__10, 104)_ = 4.37, p= 0.000). Positive significant ALP-related predictors of post-test scores were improvement after activities (β= 0.507, t_(__104)_ = 2.101, p= 0.038), timely completed objectives (β= 0.391, t_(104)_ =2.418, p= 0.017, and number of revisions (β= 0.127, t_(104)_ = 3.240, p= 0.002). Number of total activities, regardless of learning improvement, negatively predicted post-test scores (β= - 0.088, t_(__104)_ =- 4.447, p= 0.000). The significant R^2^ change following the addition of gender, GPA, and pre-test score (R^2^= 0.689, F_(__13, 101)_ = 17.24, p= 0.000), indicated that these three variables explained an additional 39% of the variance in student performance beyond that explained by ALP-related variables, which were no longer significant. Inclusion of cumulative GPA and pre-test scores showed to be the strongest and only predictors of post-test scores (β= 18.708, t _(101)_ = 4.815, p= 0.038) and (β= 0.449, t _(101)_ = 6.513, p= 0.038), respectively (Table 4).

**Table 4:** Linear regression: Final exam scores and the adaptive platform activity and outcome measures, controlling for gender and prior academic performance (n = 115)

Model	1					2				
	Unstd. Beta	Std. Beta	Part. Corr.	t	p-value	Unstd. Beta	Std. Beta	Part. Corr.	t	p- value
Knowledge state	-.302	-.065	-.049	-.504	.615	-.092	-.020	-.023	-.227	.821
Knowledge covered	1.937	.219	.147	1.280	.133	.759	.086	.086	.863	.390
Determine knowledge performed	-.136	-.208	-.169	-1.752	.083	.000	.000	.000	.003	.997
Completed objectives	-.756	-.197	-.133	-1.371	.173	-.091	-.024	-.023	-.234	.815
Timely completed objectives	.391	.283	.231	2.418	.017 ^*^	-.014	-.010	-.012	-.122	.903
Total activities	-.088	-.926	-.400	-4.447	.000 ^*^	-.011	-.113	-.071	-.715	.477
Practice operations	.067	.232	.160	1.654	.101	-.014	-.046	-.047	-.473	.637
Number of revisions	.127	.556	.303	3.240	.002 ^*^	.023	.100	.081	.817	.416
Total time	-.001	-.058	-.061	-.619	.537	.000	-.021	-.033	-.333	.740
Activities led to improvement	.507	.228	.202	2.101	.038 ^*^	.082	.037	.048	.485	.628
Gender						.910	.025	.042	.424	.672
Cumulative GPA						18.708	.373	.432	4.815	.000 ^*^
Pre-test score						.449	.511	.543	6.513	.000 ^*^
Constant	26.049				.771	-31.304				.615
Model fit	0.000					0.000				
R^2^	0.296					0.689				

* Significant P value < .05

These significant predictors, obtained from the regression models, are described next in more details. For each point increase in activities that led to learning improvement, final exam scores increased by 0.51 point. For each point increase in number of completed objectives prior to the assigned due date, final exam scored increased by 0.39 point. For each point increase in number of revisions, final exam scores increased by 0.13 point. For each point increase in number of total activities (regardless of improvement), final exam scores decreased by 0.1 point. After the addition of gender and previous academic performance, students’ GPA was shown to be the most significant predictive variable. For each point increase in cumulative GPA, final exam scores increased by 18.71 points. To a far lesser degree, pre-test score was also a significant positive predictor variable. For each point increase in pre-test scores, final exam scores increased by 0.45 point.

## Discussion

In this study, we sought to understand the impact of students learning behavior, reflected via ALP-related indicators that reflect self-regulated learning, on their learning performance, measured by final exam scores. Without controlling for background academic performance, the model with 10 ALP variables explained 29.6% of the variance in the final exam scores, and the predictability of the regression model appeared to be consistent with findings from previous studies of online learning environments (for instance, 31% in Morris and colleagues, 2005 and 33% in Macfadyen and Dawson, 2010).^15^^, ^^16^ In studies conducted in blended learning using an ALP, Chen and colleagues (2018) reported lower predictability, 25% of the variance in the cumulative grades and 16% of the variance in the final exam, when using more variables with the cumulative grade compared with final exam score, as an outcome measure.^11^ Other LMS-based studies have reported inconsistent patterns in two blended courses.^14^ Our findings may be explained, in part, by the high-stakes nature of the preparatory review course, where online homework represented a significant weight of the cumulative grade in the course.

Considering ALP-related variables solely, this study’s findings showed that three significant indicators positively impacted students’ final exam scores, including productive learning strategies, reflected by activities that lead to actual learning improvement, followed by timely submissions of ALP weekly assignments and number of revisions. On the other hand, the number of total activities performed, regardless of improvement in student’s knowledge state/level, negatively predicted students’ final exam performance. These findings, without considering students’ background academic performance, indicate that students with higher self-regulated learning skills, specifically related to effective learning strategies, self-awareness, and self-monitoring, were more likely to score higher on the final exam. In other words, successful students are those who answered questions in the ALP correctly more often, performed activities that led to actual improvement in knowledge, received and/or accessed less reading material (i.e., engaged in more concentrated study sessions), and submitted their assignments on time.

The second significant predictor found in this study was timely submission of ALP assignments, reflecting students’ time management skills or regular study behaviors by allowing a greater amount of time for reflection and synthesis. This finding is consistent with numerous studies on self-regulated learning in online learning, in which reported failure in self-regulation reflected academic procrastination, and procrastination had a significant negative impact on success in exam performance^17^^-^^19^ and potential student dropout.^4^ Further, You (2016) found that two academic procrastination indicators in LMS data explained 59.7% of the variability in students' academic achievement.^4^ In line with previous studies, this study’s findings support the perspective that time management strategies have a considerable significant impact on academic achievement and, thus, should be considered as a variable worth addressing in future updates in the ALP and/or future intervention studies.^20^^,^^21^ Given the scope of this study, we did not analyze detailed logs to objectively measure consistent study behaviors, via indicators such as frequency of accessed sessions, dates of first and last activities, and interval between activities, which is a promising area for future research, using sophisticated data mining techniques.

The final significant predictor was the number of revisions performed by students. This is an encouraging finding that reflects appropriateness of the instructional design, supported by a design-friendly adaptive system that aligns well with the nature of this preparatory review course. The system provided students with the option to revise the material after completing each learning path. Further, the course director made the learning modules available after the due date had passed. The data logs indicate that students kept reviewing material multiple times, as they continued to prepare for the NBDE, far after the course had ended. In support of this observation, Freeman and colleagues (2006) conducted a study among pharmacy students and found that 77% felt that accessing online learning materials more than once was helpful in preparing them for national exams.^22^

In the current study, inclusion of background academic data in the second prediction model impacted the significance of ALP-related predictors. Researchers have debated whether to include demographic and previous academic variables or not, the nature of inclusion and interpretation of prediction model output.^23^ Some researchers have used them as control variables,^11^ others included them as main predictors,^2^ while others did not include them.^11^ Including background information and student records significantly increases the predictive power of students’ modeling for learning and achievement,^24^ but it may be better to use them as control variables to avoid profiling students based on their inherent characteristics.^25^ Similar to this study’s findings, numerous association studies report significant strong positive correlations between cumulative GPA and academic achievement in both online and face-to-face courses.^16^^,^^26^ Also, previous predictive studies that included GPA in the regression model found GPA to be a strong predictor of post-test scores.^16^^, ^^26^

In the context of this study, initial assessment (i.e., pre-test scores) was used formatively. Yet, it presented another significant predictor of final exam performance, in comparison with other performance measures in the ALP (knowledge state and average score). This finding is consistent with results reported by other researchers who found that final exam performance can be substantially predicted by students’ performance on early assessments during the initial sections of online modules.^24^ Tempelaar and colleagues provided a rationale for this finding, stating, “That the best predictor for performance, is performance itself, will not surprise many teachers and researchers”.^24^ When considering the overall findings obtained from correlational and regression analyses, our findings contradict findings reported by McGaghie and colleagues, who suggested that deliberate practice is a more powerful predictor of professional achievement than academic aptitude or experience.^27^

As mentioned earlier, this study’s findings reinforce previous reports in the literature focused on the role of self-regulation in student success in online learning, most notably seen in time-management skills. The beneficial learning behaviors observed among successful students contradicts what prior research has found about the importance of effort with regards to achievement, known as “productive struggle”.^11^ These findings provide insight into how instructors can inform students about effective studying and learning behaviors in such a review course. While instructors should generally encourage students to “keep trying” and persist until they have learned the material, it might be more useful, at least in a context similar to this study, to stress that often efficiency is more advantageous for success than amount of effort. The study findings support this point because focused study, as derived from activities that led to improvement, was shown to correlate with success more so than “trying” and continuing to study, as derived from total activities and number of lessons. Such findings imply that not all clicks are the same. Thus, distinguishing beneficial learning behaviors from those that are less beneficial and weighting the quality rather than the quantity of learning, will support more accurate prediction of student performance and signaling of struggling students. Course instructors may encourage students to organize more concentrated study sessions, as our findings show that fewer learning sessions and overall activities resulted in better exam scores.

Nonetheless, it is important to note that efficient education and learning should not be equated to increased stress on students.^28^ As educators, we realize that student success is not entirely the result of effort and effective strategies, but encompasses other components, mainly related to students’ background knowledge level, especially in a review course like the one that was the basis for analysis in this study. Further, the negative impact of cognitive overload might have played a role in student performance, since students were trying to review as much content as possible during the last month of their preparation prior to the NBDE. These findings might be different if students were mastering new domains of knowledge, as numerous studies in education research support the perspective that greater effort leads to greater achievement.^11^

Informed by previous research, and supported by the current findings, we emphasize the importance of self-regulated learning skills in relation to academic achievement which was defined in this study by students’ performance on the final exam. In agreement with what many researchers have proposed,^4^^,^^6^^,^^11^ we believe that the elaborate data obtained from an ALP are valuable indicators for certain aspects of student learning behavior. If these indicators are interpreted carefully and contextually, the results can bring about meaningful interventions or actionable recommendations. The current study findings provide detailed insights about learning at a deeper level which cannot be generated by using only summative tests and subjective surveys. Although data interpretations of predictive model outputs are subjective, the practice and interpretation of the study findings are consistent and aligned with a theoretical learning framework, previous research, and the current study context. Involving the course instructor as a consultant figure in this study aided in validating the interpretation of study findings and potential implications. By excluding the instructor’s involvement, we would not have been able to determine his pedagogical intent.^29^

Given that the student population differs from institution to institution, the special nature of this review course and the ALP, and the predictive model obtained, our findings are specific to the context of this study and may not be directly generalizable or transferable to different contexts. Nonetheless, researchers encourage transferring the overall analytics approach across different disciplines.^6^ Although the predictive power of models, in general, has been shown to be significantly more often right than wrong in improving students' success and retention, it is important to remain cognizant of their imperfections and inaccuracies.^23^ Regarding the singularity and short duration of the 4-week review blended learning course, future studies could identify not only significant course-related measures, but also include robust measures that reflect students’ learning in numerous contexts (e.g., purely adaptive online course and different HPE disciplines) and other courses preceding this course. Further, this study would benefit from a more longitudinal analysis, beyond this course, by validating the model and identifying its predictive power in relation to future academic performance. However, the applicability of such a proposal might be challenging.

The instructional approach of the blended course included a flipped classroom design in a high-stake course where the ALP-related online assignments contributed consistently and significantly to student success in the course (30%–35% of the course grade), whereby students were required to pass the exam to pass the course and take the National Board of Dental Examination. Furthermore, the face-to-face weekly component of the course was organized sequential to what students had learned online and was intended to be provide complementary instruction utilizing small group learning approach, with more emphasis on example problems that were not mastered by students in the ALP or on content aspects that were difficult to understand (or misunderstood) by students. This study’s findings suggest that students were generally engaged with the ALP material prior to having in-class instruction time. Having said that, student self-regulation of their learning might be different in a purely online course, which is a fruitful area for future research.

The adaptive aspect of the ALP was not fully tested in this study given the proprietary nature of such information. The lack of understanding of, and detailed access to, adaptive learning systems have been described by previous researchers as major challenges to fully understanding the relationship between students’ learning patterns, characteristics, and performances within an adaptive system learning medium.^30^^, ^^31^ Finally, this quantitative study mainly depends on collection of overall averaged data, accompanied by conventional statistical analyses such as correlations and regressions. While this approach is consistent with numerous LA studies, future research could include more participants, collecting more fine-grained data, and applying more advanced statistical methods used in the field of data mining. In doing so, we will be able to process even "bigger data" and obtain a better perspective about the relationship among students’ learning behaviors, their traits and characteristics, and their performances in the domain of adaptive learning.

Finally, SRL is a complex construct and as with any complex system, online learning comprised various interacting parts that impact one another to produce specific learning outcomes for individual students. While we attempted to contextually operationalize SRL appropriately, we cannot undoubtedly state how much the ALP reflected the behaviors we interpreted. More so, we could not essentially discern whether students were engaged in meaningful productive learning processes or whether they were not actually learning, but rather involved in meaningless pauses, ranging from viewing/downloading other resources, to browsing the internet, to taking a nap, etc. This limits our ability to comprehensively understand why we observed the relationships we interpreted in our study, which present a valuable opportunity to conduct qualitative analyses as an important area for future research.

## Conclusions

Track data obtained from an adaptive learning platform can be valuable indicators of learning behavior. This study highlights the importance of effective and/or efficient learning behavior reflected by productive learning activities and time management skills. Careful and contextual analysis of students learning behavior can guide future studies to examine targeted, practical, and scalable interventions via redesigning courses and including various adaptive tools as a scaffold to support individualized learning and targeted feedback. Such adaptive interventions need to be further explored and assessed as we are only at the infancy of comprehensively understanding the adaptivity and adaptability of different systems across different contexts and how impactful they are, especially at a scalable level.

### Conflict of Interest

The authors declare that they have no conflict of interest.
